# Age-Dependent Terminal Declines in Reproductive Output in a Wild Bird

**DOI:** 10.1371/journal.pone.0040413

**Published:** 2012-07-06

**Authors:** Martijn Hammers, David S. Richardson, Terry Burke, Jan Komdeur

**Affiliations:** 1 Behavioural Ecology and Self-organization, Centre for Ecological and Evolutionary Studies, University of Groningen, Groningen, The Netherlands; 2 Centre for Ecology, Evolution and Conservation, School of Biological Sciences, University of East Anglia, Norwich, United Kingdom; 3 Department of Animal and Plant Sciences, University of Sheffield, Sheffield, United Kingdom; 4 Nature Seychelles, Victoria, Mahé, Seychelles; California State University Fullerton, Unites States of America

## Abstract

In many iteroparous species individual fitness components, such as reproductive output, first increase with age and then decline during late-life. However, individuals differ greatly in reproductive lifespan, but reproductive declines may only occur in the period just before their death as a result of an age-independent decline in physiological condition. To fully understand reproductive senescence it is important to investigate to what extent declines in late-life reproduction can be explained by age, time until death, or both. However, the study of late-life fitness performance in natural populations is challenging as the exact birth and death dates of individuals are often not known, and most individuals succumb to extrinsic mortality before reaching old age. Here, we used an exceptional long-term longitudinal dataset of individuals from a natural, closed, and predator-free population of the Seychelles warbler (*Acrocephalus sechellensis*) to investigate reproductive output, both in relation to age and to the time until the death of an individual (reverse-age approach). We observed an initial age-dependent increase in reproductive output that was followed by a decline in old age. However, we found no significant decline in reproductive output in the years directly preceding death. Although post-peak reproductive output declined with age, this pattern differed between terminal and non-terminal reproductive attempts, and the age-dependence of the terminal breeding attempt explained much of the variation in age-specific reproductive output. In fact, terminal declines in reproductive output were steeper in very old individuals. These results indicate that not only age-dependent, but also age-independent factors, such as physiological condition, need to be considered to understand reproductive senescence in wild-living animals.

## Introduction

Survival and fecundity are important fitness components that should be integrated to estimate fitness [1]. Life history decisions may affect fitness; for example, individuals may trade off early-life fecundity against future reproduction or survival, and individuals may show different age-specific schedules of reproduction [1], [2]. Understanding how and why the fitness performance of individuals changes with age is essential for understanding the evolution of life histories [1], [3]. In long-lived animals, such as mammals and birds, reproductive output generally increases with age in young individuals, and may be followed by a decline in old individuals [4]–[6]. Such changes in age-specific traits may occur within individuals, for example because of accumulated breeding experience, changes in physiology or reproductive allocation [7]. Although the causes and patterns of initial improvement of reproductive performance with age are reasonably well studied, at least in avian species [7], [8], relatively little is as yet known about ageing patterns and decreases in reproduction in older individuals [9], [10]. Such late-life declines in reproductive performance may be caused by senescence, defined as the progressive deterioration of an individual's physiological and cellular function with age [11], [12]. Alternatively, a decrease in reproductive allocation at older ages in order to maintain survival may generate a pattern similar to senescence [13]. Until recently it was thought that high extrinsic mortality (e.g. predation, disease) prevented individuals from living long enough to show senescence in wild populations [12]. However, there is accumulating evidence that senescence is an important part of the life-history of animals in the wild [9], [10], [14].

To date, the majority of studies that have investigated reproductive senescence in the wild have studied age-dependent changes in reproductive traits [6], [15]–[18]. However, reproductive performance may also be influenced by factors that are independent of the age of an individual, for example when reproductive performance is better explained by physiological condition than by chronological age. Because the death of an individual can be seen as the outcome of the accumulation of physiological damage over time, and because the rate of damage accumulation differs between individuals, the time until the death of an individual may reflect physiological condition and reproductive performance better than chronological age [10], [13], [19]. Indeed, individuals often differ greatly in reproductive lifespan or breeding experience, but may only show signs of reproductive senescence close to their death [10], [20], [21].

Unfortunately, accurate data on individual birth and death dates are lacking in many study systems, which hampers the investigation of age-independent terminal declines in reproductive output. Some evidence for the contribution of age-independent factors to reproductive senescence comes from studies that showed that early-life investment and conditions are associated with reproductive performance during late-life [2], [16], [22], [23]. Since such factors affect physiological condition rather than chronological age, these studies suggest that reproductive senescence contains age-independent components. Furthermore, evidence that both age-dependent and age-independent effects may co-occur and affect reproductive senescence comes from a study on bighorn ewes (*Ovis canadensis*). Female reproductive output showed an age-independent terminal decline, and this terminal decline was steeper for older individuals [19]. Therefore, to understand senescence it is important to investigate the relative importance of both age-dependent and age-independent components [19].

To study senescence in the wild, long-term, longitudinal datasets of marked animals living in closed populations with low levels of extrinsic mortality and repeated observations of individuals from birth to death are required (see [9]). In this study we use the long-term dataset (1997–2010) on the Cousin Island population of the facultatively cooperative Seychelles warbler (*Acrocephalus sechellensis*) to study patterns of late-life age-dependent reproductive performance. In this population, the majority (>96%) of individuals have been individually colour-marked [24] and there is virtually no inter-island dispersal [25]. This lack of dispersal provides a rare opportunity to follow the reproductive performance of all individuals within a single population from birth to death. Importantly, predation on adults is absent, so individuals are expected to live long (life expectancy at fledging = 5.5 years [26], maximum lifespan = 17 years). Indeed, a previous, largely cross-sectional, study of a single cohort showed that reproductive output declined in old Seychelles warblers of both sexes [27]. The Seychelles warbler is therefore extremely suitable to study late-life age-specific reproductive performance in the wild.

Here we investigated patterns of within-individual late-life reproductive performance in female Seychelles warblers. We focussed primarily on females since estimating male reproductive success is confounded by the high incidence of extra-group paternity (40% [28]). In addition, co-breeding and helping behaviour of subordinates may influence the reproductive success of the breeding pair [29], [30]. Therefore, we adopted a conservative approach and focussed on breeding attempts by the majority of socially monogamous females that were not assisted by helpers.

We investigated reproductive performance both in relation to birth and death (reverse-age approach). This is important because such an analysis allows us to establish whether late-life declines in reproductive output are age-dependent, age-independent, or both. As individuals vary in intrinsic quality, life history and the environmental conditions they face [31], [32], their physiological condition may change independently of chronological age. Hence, individuals may senesce and die at different chronological ages, and age-independent late-life decreases in physiological condition may increase late-life age-dependent reproductive declines [19]. Age-dependence occurs if late-life declines in reproductive output are only related to chronological age, but not to the time until an individual's death. Age-independence occurs when such declines are related to the time until death, but not to chronological age. If reproductive output declines close to the death of an individual, but only in very old individuals, both age-dependent and age-independent factors explain reproductive senescence.

## Methods

### Ethics statement

All fieldwork performed complied with local ethical regulations and agreements. The Seychelles Department of Environment and the Seychelles Bureau of Standards gave permission for fieldwork and sampling. Nature Seychelles allowed us to work on Cousin Island Nature Reserve.

### Study population and data collection

The Cousin Island (29 ha, 4°20′ S, 55°40′ E) population of Seychelles warblers, which at carrying capacity comprises *ca* 320 colour-banded individuals of known age in *ca* 115 territories [33], has been monitored since 1981 as part of a long-term study [34]. The warbler's life history is characterised by high annual adult survival (84%), mostly single-egg clutches, and extended periods of parental care (3–6 months, [27], [31]. Seychelles warblers are almost completely insectivorous and territory quality (measured in terms of insect prey availability in a territory) is important for reproductive success [35]. Reproduction is seasonal with most birds (annual mean = 93.8% of territories) breeding in the southeast monsoon season (June–September, hereafter: “main breeding season”) when food availability is high. A smaller fraction of the population (annual mean = 47.8% of territories) additionally attempts to breed during the northwest monsoon season (January–March, hereafter: “minor breeding season”) [36]. Reproductive success during this period is generally very low, as only about half of the nests reach the clutch stage, and only 20% of these produce fledglings [37]. Although Seychelles warblers can breed successfully in socially monogamous pairs, cooperative breeding occurs frequently [35] and is primarily driven by the shortage of breeding vacancies [35], [38]. An individual's first independent breeding may occur between one and eight years of age (this study). Individuals that have acquired a breeding position will typically remain on and defend the same stable territory until their death, or until they are demoted from their dominant breeding position [30].

During the main breeding season in each year from 1997–2010 we checked each territory for breeding activity at least once every two weeks by following the resident female for 30 minutes [30]. Once nest building commenced, all territories and nests were visited every three to four days for at least 10 minutes to monitor the progress of each breeding attempt. All breeding attempts were followed at least until the nestling fledged (18–20 days after hatching [26], or until the breeding attempt failed. After fledging, fledglings can be easily observed as they are begging for food almost continuously for several weeks, and remain with their parents in their natal territory for several months [26].

### Data selection and definition of variables

Breeding success was defined as the production of a fledgling during the main breeding season. Although the minor breeding season may provide interesting information with respect to the timing of age-dependent breeding attempts, we excluded them from our analyses, because there is much less reproductive activity during this period and consequently they are not monitored as thoroughly. Consequently we do not have sufficient data to comprehensively evaluate differences in age-dependent reproductive success between the two breeding seasons. However, a preliminary cross-sectional analysis, using two years for which data on reproductive success during the minor breeding season was available, suggests that females show a similar age-specific pattern of reproductive success during this period as reported in this study (Age: β±SE  = 0.12±0.09, Age^2^: β±SE  = −0.05±0.03; M. Hammers, unpublished data). Except for 2004, data on breeding success during the main breeding season was available for all individuals in all breeding groups. This includes individuals that were born and colour-banded before this period (since 1985), but that were dominant breeders within this period. As the annual resighting probability of dominant breeding individuals in this population is virtually one (0.98±0.01 SE [24]), and because inter-island dispersal is extremely rare [25]), we could safely assume that individuals that were not seen the next year were dead. We only considered the breeding success of females that were not assisted by helpers. In virtually all cases, these non-cooperatively breeding females always produced 0 or 1 fledglings per breeding attempt. We only included females that were known to have died before the end of the study period, thus for all individuals both the birth and death year were known. Importantly, the entire population of non-cooperatively breeding females (*ca* 80% of all territories) was followed every year. For each female the age of the first reproductive attempt as dominant breeder (AFR) and the age of the last reproductive attempt as dominant breeder (ALR, range: 1–16) were recorded. The majority of females (244/283 individuals) started breeding in their second or third year of life, the few females that bred after this (in or after their fourth year of life) were in lumped into one group.

### Statistical analyses

Factors such as territory quality and the presence of any recent offspring (i.e. offspring born during the minor breeding season) within the territory may affect reproductive output independent of age or individual quality. Recent offspring may affect reproductive output of the dominant breeders due to food competition [39]. We statistically controlled for these factors by including them as covariate (territory quality) or binary factor (presence of recent offspring Y/N) in the models. A territory quality index was calculated following Komdeur [35] and Van de Crommenacker et al. [40] using the formula *a* • ∑ (*c_x_ • i_x_*) where *a* is the territory size in hectares, *c_x_* is the foliage cover for broad-leafed tree species *x*, and *i_x_* is the mean monthly insect count for tree species *x* per unit leaf area (dm^2^). Territory size was determined from detailed observational data of foraging and territorial defence behaviour. Foliage cover was scored by determining the presence of each tree species at 20 random points in every territory on the island, in the following height bands: 0 to 0.75 m, 0.75 to 2 m and at 2 m intervals thereafter. Insect counts were estimated by monthly counting the total number of insects on the undersides of 50 leaves for each tree species present in 14 different regions across the island. Because territory quality was not measured every year we calculated one index of relative territory quality for each territory, rather than extrapolating territory quality for the missing years. To this end, we calculated the standardized territory quality for each territory in each year (mean-centred by year and divided by the standard deviation), and averaged these values to obtain one value of quality for each territory for all years combined (territory quality, Table 1). When we repeated the analyses using non-standardized territory quality, for the years for which data were available, the results remained the same. In addition, for each year we included the mean number of fledglings produced by all non-cooperatively breeding females as an additional measure for territory quality, assuming that the average number of fledglings is higher in years when food availability is high. For all analyses, we used generalized linear mixed models (GLMM) with a binomial error structure and a logit link function. Because of the non-independence of repeated observations of the same female and large differences in reproductive success between years, we included female identity and year as cross-classified random effects. Such mixed models, in which a random intercept is assigned for each individual, can be used to investigate within-individual patterns of age-specific reproductive performance [41]. We investigated within-individual effects of age (“age model”) and years before death (“YBD model”), and included AFR and ALR to control for the appearance or disappearance of individuals [41]. We included a binary factor to categorize each breeding attempt as being the terminal reproductive attempt or not, to investigate whether reproductive output during the terminal reproductive attempt was lower (see [15]).

**Table 1:** Definition of the variables considered in the analyses.

Variable	Definition
Age	Chronological age of each female (year of the breeding attempt – birth year)
YBD	Number of years before the death of the female (death year – year of the breeding attempt)
Year quality	Average number of fledglings produced by all non-cooperatively breeding females in the year of the breeding attempt
Territory quality	Average standardized territory quality of the female's resident territory, relative to all other territories
OFL present	Presence of an independent fledgling from the minor breeding season, as an indication of recent reproduction
AFR	Age of first independent breeding
ALR	Age of last independent breeding
Terminal	Binary factor categorizing each breeding attempt to be the last or not

First, we investigated whether the quadratic effect of age (age^2^) and YBD (YBD^2^) were associated with reproductive output after centring age and YBD on the mean (to reduce collinearity between the linear and quadratic terms). A negative coefficient of the quadratic term would suggest an initial increase in reproductive output, followed by a decline. To confirm the fit of, and the reproductive decline suggested by, the quadratic model, we repeated the analyses using generalized additive mixed models (GAMM) with a non-parametric smoothing spline for age to replace the linear and quadratic effects of age. In addition, we used a piecewise regression with one threshold age. Here, two linear regression slopes are fitted on both sides of a focal age. To select the threshold age that best matches the data, all possible threshold ages were considered and the threshold age with the lowest AIC value (a measure of the relative goodness of fit of the model) was selected. We fitted the piecewise regression models without random effects, because the models did not converge when random effects were included. Second, we investigated whether reproductive output showed a significant linear decline after the age at which reproductive output is highest. Subsequently we investigated whether this pattern was different for the terminal breeding attempt by investigating the interaction between age (age^2^) and the terminal breeding attempt. Following Martin & Festa-Bianchet [19], a significant interaction would suggest that an age-independent component varies with age.

We performed the analyses using R (version 2.12.2 [42]). The GLMMs were fitted using the Laplace approximation with the package lme4 (version 0.999375 [43]), and the GAMMs using the package mgcv (version 1.7–4 [44]). We checked for overdispersion in the data by estimating the dispersal parameter, but did not find evidence for this (dispersal parameter close to one). All two-way interactions between age (or YBD) and AFR, ALR and terminal reproductive attempt were tested, but only reported when statistically significant (P<0.05) or of particular interest. Non-significant terms were sequentially (in order of least significance) removed from the models, starting with interaction terms. Main effects associated with significant interaction terms were not removed from the models. The significance of main effects was assessed after refitting the model without the associated interactions terms. Table 1 contains a brief description of all explanatory variables that are used in the models.

## Results

### Age-dependent reproduction – all ages

**Figure 1:**
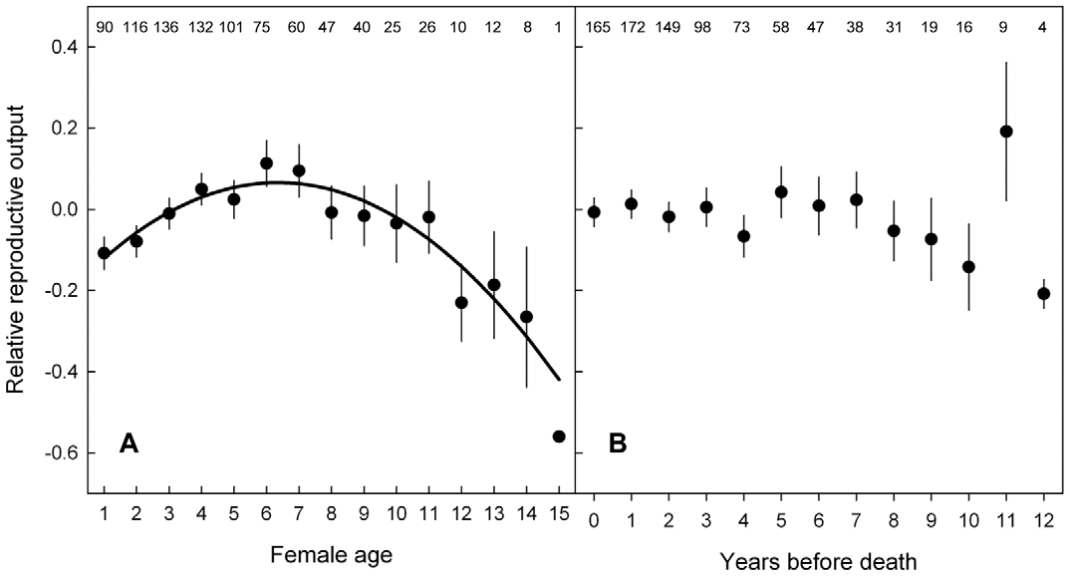
Reproductive output in relation to chronological age and the number of years before death (YBD). Relative reproductive output is the number of fledglings produced by a female minus the averaged reproduction of all females in a given year. Data are means and standard errors. Sample sizes are given for each age and YBD class. The solid black line in panel **A** shows the within-individual pattern of age-specific reproduction from the final model from table 2a, except that this model was fitted without interaction terms. There was no relationship between reproductive output and YBD (panel **B**, see table 2b).

Reproductive success showed a clear age-related pattern, with an initial increase until a peak between 6 and 7 years of age and thereafter a decline (Table 2a, Fig. 1a). The non-parametric smoothing function for age from the GAMM showed a very similar pattern compared to the quadratic age effect as shown in Table 2a and Fig. 1a (peak between 6 and 7 years; smoothing function for age significant with an estimated degrees of freedom of 2.9, F = 7.64, p<0.001). The piecewise regression model that was best supported by the data indicated a threshold age of 7 years (AIC  = 1016.32), although the models with a threshold age of 6 or 8 years were very similar (ΔAIC  = 0.06 and ΔAIC  = 0.78, respectively). Models with other threshold ages were less supported by the data (threshold age 5, ΔAIC  = 2.63; all other threshold ages, ΔAIC >4.22). These models had a positive slope before and a negative slope after the threshold age.

**Table 2:** Linear mixed models of fledgling production (yes/no) in relation to (A) age and (B) years before death (YBD).

A	Age models				B	Years before death models			
	Estimate	SE	z	P		Estimate	SE	z	P
**Intercept**	−2.28	0.24	−9.67	<0.001	**Intercept**	−2.44	0.22	−10.90	<0.001
**Age**	0.10	0.04	3.34	0.001	YBD	−0.01	0.03	−0.34	0.734
**Age^2^**	−0.03	0.01	−4.63	<0.001	YBD^2^	0.00	0.01	−0.05	0.962
**Year quality**	4.96	0.60	8.25	<0.001	**Year quality**	4.86	0.57	8.46	<0.001
**Territory quality**	0.37	0.15	2.42	0.016	**Territory quality**	0.36	0.15	2.42	0.016
OFL present	−0.35	0.22	−1.57	0.117	OFL present	−0.21	0.22	−0.98	0.329
AFR	−0.18	0.12	−1.52	0.128	AFR	−0.10	0.12	−0.84	0.400
ALR	−0.01	0.04	−0.29	0.774	ALR	0.01	0.02	0.27	0.791
**Terminal**	0.45	0.26	0.09	0.929	Terminal	−0.10	0.23	−0.43	0.667
Terminal * Age	0.11	0.09	1.14	0.256					
Terminal * Age^2^	−0.05	0.02	−2.43	0.015					
	Variance	SD				Variance	SD		
**Female (random)**	0.11	0.33			**Female (random)**	0.09	0.30		
**Year (random)**	0.00	0.00			**Year (random)**	0.00	0.00		

Summaries were derived from binomial response, linear mixed models (see Methods for details). Variables included in the final models are indicated in bold. The models consisted of 879 breeding attempts by 283 dominant females of all ages. Abbreviations of explanatory variables are explained in table 1.

**Figure 2:**
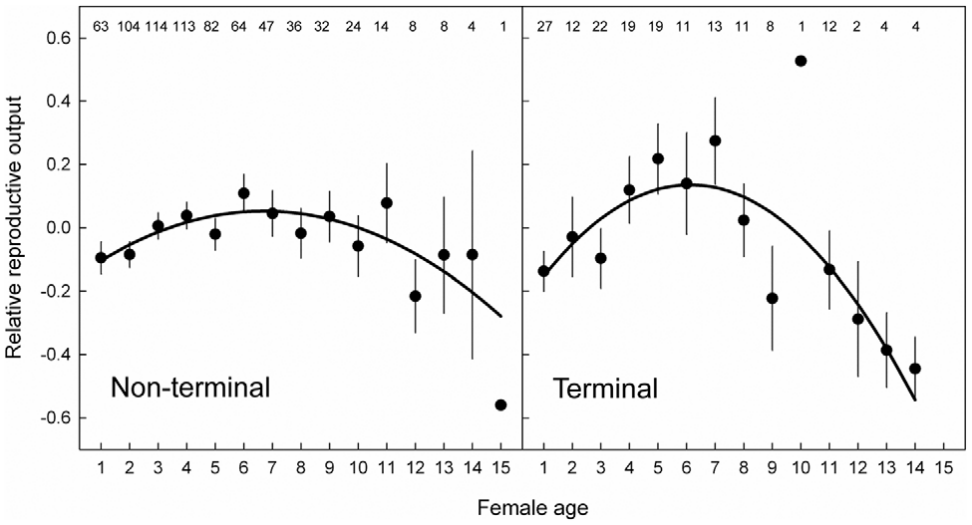
Age-dependent terminal declines in reproductive output. Age-specific relative reproductive output in relation to whether a reproductive attempt was the last (“Terminal”, right panel), or not (“Non-terminal”, left panel). Relative reproductive output is the number of fledglings produced by a female minus the averaged reproduction of all females in a given year. Data are means and standard errors. Sample sizes are given for each age class. The solid lines are the model predicted regression slopes from the final model from table 2a. The late-life decline in reproductive output was steeper during terminal reproductive attempts (see table 3a).

There was no association between AFR or ALR and reproductive success (Table 2a). The pattern of age-specific reproduction appears to be different for the terminal breeding attempt compared to non-terminal breeding attempts with a lower reproductive output in the terminal breeding attempt in young and old individuals and a higher reproductive output in middle-aged individuals (Table 2a, Fig. 2). We found no evidence for any relationship between YBD and reproductive success (Table 2b, Fig. 1b).

**Table 3:** Linear mixed models of fledgling production (yes/no) in relation to (A) age and (B) years before death (YBD) after the peak in reproductive output.

A	Age models				B	Years before death models			
	Estimate	SE	z	P		Estimate	SE	z	P
**Intercept**	−2.13	0.45	−4.70	<0.001	**Intercept**	−0.08	0.90	−0.09	0.930
**Age**	−0.11	0.10	−2.74	0.006	YBD	0.13	0.12	1.09	0.275
**Year quality**	3.79	1.14	3.34	<0.001	**Year quality**	3.28	1.11	2.96	0.003
**Territory quality**	0.55	0.34	1.59	0.112	Territory quality	0.45	0.32	1.40	0.162
OFL present	−0.88	0.49	−1.78	0.074	OFL present	−0.71	0.48	−1.48	0.140
AFR	−0.31	0.26	−1.20	0.232	AFR	−0.26	0.25	−1.04	0.300
ALR	−0.05	0.11	−0.42	0.676	**ALR**	−0.17	0.07	−2.32	0.020
**Terminal**	−0.18	0.43	−0.27	0.789	Terminal	−0.67	0.41	−1.63	0.102
Terminal * Age	−0.56	0.24	−2.36	0.018					
	Variance	SD				Variance	SD		
**Female (random)**	0.83	0.91			**Female (random)**	0.55	0.74		
**Year (random)**	0.00	0.00			**Year (random)**	0.04	0.20		

Summaries were derived from binomial response, linear mixed models (see Methodsfor details). Variables included in the final models are indicated in bold. The models consisted of 229 breeding attempts by 90 females that were older than 6 years. Abbreviations of explanatory variables are explained in table 1.

### Age-dependent reproduction – individuals older than six years

The peak reproductive output was between 6 and 7 years of age (see above). Therefore we investigated the decline in reproductive output with age for females older than 6 years (229 breeding attempts of 90 females). There was a significant decline in reproductive output with age (Table 3a, parameter estimate of age of a model that was fitted without interaction terms: β = −0.24, SE  = 0.09). Again, the age-dependent decline was sharper for the terminal attempt compared to all other breeding attempts, as indicated by the significant “age * terminal” interaction (Table 3a, Fig. 2). In other words, the decline in reproductive output was steeper for older individuals. We repeated this analysis and replaced the age effects with ALR. This confirmed that birds with longer reproductive lifespans showed greater terminal declines in reproductive output (interaction ALR * terminal: β = −0.56, SE  = 0.23, z = −2.42, p = 0.016). Interestingly, the decline in reproductive output with age after the predicted peak in reproductive output appears to be explained mainly by the age-dependence of the terminal reproductive attempt (see Fig. 2). Indeed, when we repeated the final model from Table 3a on the same dataset without terminal reproductive attempts, the parameter estimate of age was no longer significant (β = −0.10, SE  = 0.10, z = −1.04, p = 0.297). There was no relationship between reproductive output and YBD (Table 3b). In addition, the interaction between YBD and ALR was not significant (β = <0.01, SE  = 0.04, z = 0.11, p = 0.912).

## Discussion

We demonstrated that female Seychelles warblers show a clear within-individual change in reproductive output in relation to chronological age, but not in relation to the time until death. Reproductive output increased until seven years of age and decreased thereafter. This age-dependent pattern is more pronounced during the terminal reproductive attempt compared to non-terminal attempts, which showed less age-dependence. Importantly, as the terminal reproductive attempt itself is age-dependent, this may explain much of the observed variation in age-specific reproductive output. Furthermore, this age-dependence of the terminal reproductive attempt suggests that both age-dependent and age-independent factors may play a role.

### Age-dependent terminal declines

Until recently it was believed that individuals in the wild do not senesce because most individuals die of extrinsic causes, such as predation or disease [12]. However, there is accumulating evidence that late-life declines in reproductive output do make up an important part of the life-history of animals in wild populations [15], [17]–[19], [45]–[48]. In Seychelles warblers these late-life declines in reproductive output may be common because of the atypical lack of predation on adults and the relatively stable breeding environment.

A lower reproductive performance during the last reproductive attempt(s), regardless of the age of the individual, may, at least in theory, generate apparent gradual declines in reproductive output in cross-sectional analyses, but may also confound within-individual patterns. Such terminal declines in reproductive output may be caused by an illness [21] or by a deteriorated physiological condition close to death [13], which are expected to occur independently of age [10], [13]. At least three studies have shown that terminal reductions in reproductive performance do occur and have stressed the importance of considering such effects [20], [21], [49]. Alternatively, declines in late-life performance may be more consistent with a gradual decline, rather than just a worse performance during the terminal attempt [23], [47], [49]. In our study, post-peak reproductive output declined with age, but this pattern differed between terminal and non-terminal reproductive attempts. In fact, it appeared that the terminal reproductive attempt itself was age-dependent, with terminal declines in reproductive output being much steeper in older individuals. If post-peak declines in reproductive output were entirely age-dependent, we would expect these declines to be related to chronological age, and not to the time until the death of an individual. Conversely, if these post-peak reproductive declines were entirely age-independent, for example if they result from poor physiological condition just prior to death, we would expect such declines only close to the death of an individual, regardless of age. However, we found that post-peak declines in reproductive output are best explained by a combination of both chronological age and the time until death, which suggests that both age-dependent and age-independent factors co-occur and explain reproductive senescence.

For example, physiological condition may, independent of age, decline just before death, but this may most severely affect reproductive output in very old individuals that already suffer from an age-dependent reduced general performance (e.g. foraging performance [46], [50]). A similar age-dependence of the terminal reproductive attempt(s), and hence combination of age-dependent and age-independent components, was found in a study on bighorn ewes, except that the age-dependent decline occurred during the last two years of life [19]. As reproductive output can be viewed as the outcome of several age-related factors, including reproductive allocation, and physiological condition, we can only speculate about the possible causes of the age-dependence of the terminal breeding attempt. One possible scenario is that if individuals are able to assess that the current reproductive attempt may be their last, for example because they are ill, they should invested more resources in this attempt [13], [51]. Under such a scenario, prime-aged birds may be able to compensate for a terminal illness by increasing their terminal reproductive investment. However, young birds with little breeding experience, or old birds with deteriorating physiological condition, may not be able to compensate by investing more in reproduction, leading to a reduced reproductive output during their terminal reproductive attempt (see [49]). Future studies may experimentally investigate this possibility by subjecting individuals of different ages to an immune challenge [51].

### Late-life reproductive output in relation to years before death

Late-life declines in reproductive performance are thought to be caused by physiological senescence [9], or by declines in reproductive allocation [13]. One may argue that, because individuals of different quality may age at different rates, late-life reproductive declines should occur during the few years before death, rather than in relation to chronological age only. Few studies have looked at patterns of age-specific reproduction in relation to the years before disappearance from the population, or the end of the reproductive career (e.g. [2], [19], [47], [52]. Reed et al. [2] showed that the reproductive output of common guillemots (*Uria aalge*) declined during the three years preceding disappearance from the population, but only in individuals older than 8 years. The reproductive output during terminal breeding attempts was much lower compared to all other breeding attempts, which may suggest that terminal illness plays an important role in this case (see [10]). In that study no direct comparison of reproductive output in relation to chronological age and years before disappearance was made [2]. McCleery et al. [52] showed that laying date and clutch size of mute swans (*Cygnus olor*) showed clear age-dependence, but the patterns of laying date and clutch size differed in relation to chronological age, and time until last reproduction. Increases in laying date and declines in clutch size only occurred in individuals >12 years old; laying date increased during the last few reproductive attempts, whereas clutch size only appeared to decline during the terminal reproductive attempt [52]. In contrast, Dugdale et al. [47] showed that in badgers (*Meles meles*), patterns of late-life declines in reproductive output were gradual and similar for both chronological age and time until last reproduction. Similar to our study, in a study on bighorn ewes, Martin & Festa-Bianchet [19] found no clear relationship between YBD and reproductive output in bighorn sheep, but showed an age-dependent decline during the last two years of life. The lack of a clear relationship between reproductive output and YBD in Seychelles warblers may be because individuals show a decline in reproductive output just during the terminal reproductive attempt, rather than during the last few reproductive attempts. In addition, in our study, any relationship between reproductive output and YBD may be masked by the age-dependence of the terminal reproductive attempt.

### Concluding remarks

Our study shows that patterns of age-specific reproduction may differ considerably with respect to chronological age and years before death and between terminal and non-terminal breeding attempts. Furthermore, age-dependent and age-independent reproductive senescence may interact. A similar age-dependence of the terminal reproductive attempt has been shown to occur in mammals [19], therefore we suggest that this may be a more general, and until recently overlooked, phenomenon across animal groups.
